# Biodegradable Silk Fibroin Matrices for Wound Closure in a Human 3D Ex Vivo Approach

**DOI:** 10.3390/ma17123004

**Published:** 2024-06-19

**Authors:** Jan Tinson Strenge, Ralf Smeets, Fateme Nemati, Sandra Fuest, Sophie Charlotte Rhode, Ewa Klara Stuermer

**Affiliations:** 1 Department of Oral and Maxillofacial Surgery, University Medical Center Hamburg-Eppendorf, 20246 Hamburg, Germany; j.strenge@uke.de (J.T.S.); r.smeets@uke.de (R.S.); 2 Department of Oral and Maxillofacial Surgery, Division of Regenerative Orofacial Medicine, University Medical Center Hamburg-Eppendorf, 20246 Hamburg, Germany; fateme.nemati@tuhh.de (F.N.); s.fuest@uke.de (S.F.); 3 Institute of Bioprocess and Biosystems Engineering, Hamburg University, 21073 Hamburg, Germany; 4 Department of Plastic, Reconstructive and Aesthetic Surgery, University Medical Center Hamburg-Eppendorf, 20246 Hamburg, Germany; s.rhode@uke.de; 5 Department for Vascular Medicine, Translational Wound Research, University Medical Center Hamburg-Eppendorf, 20246 Hamburg, Germany

**Keywords:** silk biomaterials, silk fibroin, wound healing, skin wounds, human skin ex vivo culture, keratinocyte migration

## Abstract

In this study, the potential of silk fibroin biomaterials for enhancing wound healing is explored, focusing on their integration into a human 3D ex vivo wound model derived from abdominoplasties. For this purpose, cast silk fibroin membranes and electrospun nonwoven matrices from *Bombyx mori* silk cocoons were compared to untreated controls over 20 days. Keratinocyte behavior and wound healing were analyzed qualitatively and quantitatively by histomorphometric and immune histochemical methods (HE, Ki67, TUNEL). Findings reveal rapid keratinocyte proliferation on both silk fibroin membrane and nonwoven matrices, along with enhanced infiltration in the matrix, suggesting improved early wound closure. Silk fibroin membranes exhibited a significantly improved early regeneration, followed by nonwoven matrices (*p* < 0.05) compared to untreated wounds, resulting in the formation of multi-layered epidermal structures with complete regeneration. Overall, the materials demonstrated excellent biocompatibility, supporting cell activity with no signs of increased apoptosis or early degradation. These results underscore silk fibroin’s potential in clinical wound care, particularly in tissue integration and re-epithelialization, offering valuable insights for advanced and—as a result of the electrospinning technique—individual wound care development. Furthermore, the use of an ex vivo wound model appears to be a viable option for pre-clinical testing.

## 1. Introduction

Wound regeneration is a complex, well-orchestrated physiological process. Depending on the wound size and regenerative capacity, a sequential healing process begins, varying in duration [1]. Widely accepted for wound care, wound matrices protect against infection and dehydration [2]. However, after wound bed conditioning, the final step of wound healing, epithelialization, often takes considerable time, so it would be beneficial to have a tool or matrix that supports this step and does not need to be removed. Therefore, optimal wound care utilizes biocompatible materials that not only enhance healing and degrade appropriately but also prevent contamination and are easy to use and access. Traditional treatments, often costly, invasive, or harmful, fall short of these ideals [3,4]. Commonly used biomaterials like collagen, gelatin, chitosan, and alginate are known for their natural occurrence and regenerative properties. However, challenges such as processing difficulties, structural degradation, and immunogenicity limit their full potential, prompting research into new biomaterials [4].

Among these, silk fibroin stands out in biomedical engineering for its unique wound-healing properties. Derived from *Bombyx mori* silkworm cocoons and the webs of spiders or other insects [5], silk fibroin, a fibrous protein, is often isolated for therapeutic use. At the same time, sericin, potentially immunogenic, is removed [6]. Once regenerated and further processed via methods like electrospinning, silk fibroin’s versatility makes it valuable in various wound care applications, from scaffolds to drug delivery systems [4,7]. Most studies have relied on animal models or 2D in vitro cultures [8,9,10], with only a few conducting clinical trials [11,12]. As regulations on animal studies evolve and their costs rise, alternative models like human skin explants offer a more advantageous approach for pre-animal testing evaluations [13]. While reconstructed skin models, comprising keratinocytes and fibroblasts, have limitations due to their lack of complete three-dimensional organization and certain cell types [14], human skin models, closely mimicking in vivo repair, are crucial for exploring clinical and biomechanical aspects of skin healing. Therefore a 3D ex vivo wound model using human skin explants, similar to the approach of Wilkinson et al. [15] was employed in this translational study. Different studies have highlighted the model’s viability for dermal healing research and emphasized the effectiveness of evaluation in wound closure [13,16,17,18].

Silk fibroin scaffolds, notable for their tunable mechanical properties, can be optimized for cell differentiation and other biomedical applications [19]. Their adaptability, especially when blended with materials like chitosan, creates composite scaffolds enhancing wound healing [4,20,21]. The biocompatibility and controlled degradation of silk fibroin scaffolds are particularly advantageous for tissue regeneration [22,23]. The degradation of silk materials, influenced by processing methods, concentration, and structure, varies; in vitro, they can degrade within three weeks [24]. An in vivo study shows different degradation rates, with aqueous-derived scaffolds beginning to degrade within two weeks and nearly complete degradation by 6–12 months, while those from organic solvents like hexafluoro isopropanol (HFIP) maintain structure for a longer period. The immune cell-mediated response accelerated scaffold degradation, leading to structural loss within 8 weeks [22]. These dynamics provide a versatile tool for selecting appropriate scaffolds for wound care. Given the ongoing search for effective wound care solutions, silk fibroin-based materials represent a promising avenue, merging the benefits of natural polymers with biomedical engineering precision [4,10,25,26]. 

In this study, our primary objective was to provide a comprehensive evaluation of the regenerative potential of silk fibroin in facilitating wound healing using a 3D ex vivo model. Specifically, we investigated the efficacy of biodegradable fibroin materials, such as cast fibroin membranes and electrospun nonwovens, in the treatment of partial-thickness wounds in human skin explants. Our focus was to assess the effect of these materials on critical aspects of wound healing, such as tissue integration and re-epithelialization. Over a controlled incubation period of 5 to 20 days, we systematically analyzed key indicators of cellular proliferation and apoptosis.

An advanced feature of this research is the use of a 3D ex vivo human skin model, which closely mimics in vivo conditions and provides a more accurate representation of the human skin wound healing processes compared to traditional animal models or 2D in vitro cultures. This innovative approach allows a more preciseevaluation of the regenerative potential of silk fibroin-based materials in a human context—as skin explant models enable the assessment of re-epithelialization in human skin with uniform wound sizes under standardized conditions [27]. By exploring the mechanisms involved in silk fibroin-mediated wound repair, our study aims to provide valuable insights that may help in the development of innovative therapeutic strategies to promote efficient and effective wound healing. This study also applies materials that resulted from advanced fabrication techniques, such as electrospinning, with enhanced properties for cell infiltration and tissue integration, demonstrating the potential for these materials to revolutionize wound care. Through this research, we aim to bridge the gap between preclinical testing and clinical application, providing a new perspective on the use of silk fibroin in wound management.

## 2. Materials and Methods

### 2.1. Silk Materials

#### 2.1.1. Preparation of Silk Solution

Silk fibroin aqueous solution was obtained using PureSilk^®^ technology (Fibrothelium GmbH, Aachen, Germany), enabling medical grade quality production of silk on an industrial scale for a broad range of concentrations. Briefly, fibroin was separated from sericin by degumming it in hot alkali solution before dissolving it in a proprietary non-toxic solvent system based on Ajisawa’s reagent [28,29,30].

#### 2.1.2. Casting of Membranes

The production of silk fibroin membranes involved silk fibroin casting according to our previous study [29,30]: The fibroin solution was cast into a polytetrafluoroethylene (PTFE) mold and left to dry for 24 h at 21 °C under a laminar flow hood, resulting in a delicate membrane with a thickness of approximately 0.1–0.15 mm. To fit them into the target wounds, disks of 3 mm diameter were created using a biopsy punch.

#### 2.1.3. Electrospinning of Nonwovens

Following the methods of our previous study [28], additive-free silk fibroin nonwovens were electrospun and directly post-treated in an ethanol bath (70 v%) for 1 h to obtain insolubility, resulting in nonwovens with a thickness of around 0.1–0.2 mm. Beta-sheet formation was acquired by immersion in 90% (*v*/*v*) aqueous methanol solution for 20 min. To fit them into the target wounds, disks of 3 mm diameter were created using a biopsy punch.

### 2.2. Biological Evaluation (Biocompatibility)

The biocompatibility evaluations were conducted partly following the procedures outlined in our earlier study [28], according to EN ISO 10993-5 [31]. This process included two key steps: First, we directly tested by incubating mouse fibroblasts (L929 cells) and human immortalized keratinocytes (HaCaT cells) on the specimen surfaces, followed by a live/dead staining assay. Second, for indirect testing, we used extracts from medium-incubated fibroin matrices. These extracts were then applied to the different cell types to assess cytotoxicity using lactate dehydrogenase (LDH) and proliferation using 2,3-bis-(2-methoxy-4-nitro-5-sulfophenyl)-2H-tetrazolium-5-carboxanilide (XTT) calorimetric activity.

#### 2.2.1. Sterilization of Specimens

All samples (membranes, nonwovens, and reference materials) were sterilized by placing them under ultraviolet light for 30 min.

#### 2.2.2. Reference Materials

Two types of reference materials were employed. The first, designated as RM-A (reference material-A), is a polyurethane film infused with 0.1% zinc diethyldithiocarbamate (ZDEC), sourced from the Hatano Research Institute in Hadano, Japan. This served as toxic control. For the negative control, particularly in the live/dead staining assay, we used tissue culture coverslips (TCC, Cat. No. 83.1840.002; Sarstedt AG & Co. KG Nümbrecht, Nümbrecht, Germany), selected for their non-toxic properties. Pure culture medium served as negative control in indirect testing.

#### 2.2.3. Cell Culture

L-929 mouse fibroblast cells were acquired from the European Collection of Cell Culture (ECACC), Salisbury, UK. These cells were cultured in minimum essential medium (MEM) enriched with 10% fetal bovine serum, 100 U/mL penicillin/streptomycin (Life Technologies Corp., Carlsbad, CA, USA), and 4 mM L-glutamine (Sigma-Aldrich Chemie GmbH, Taufkirchen, Germany), hereafter referred to as “cell culture medium”. Cultivation was conducted at 37 °C in an environment with 5% CO_2_ and 95% relative humidity. The cells were passaged upon reaching approximately 80% confluency. 

HaCaT cells, human immortalized keratinocytes, were obtained from CLS Cell Lines Dienstleistung GmbH (CLS, Eppelheim, Germany). The cells were cultivated in Dulbecco’s modified Eagle medium (DMEM; Fisher Scientific, Waltham, MA, USA) that was supplemented with 10% fetal bovine serum (FBS), antibiotics (penicillin/streptomycin; 10 µg/mL), and the growth factors EGF (epidermal growth factor, Sigma-Aldrich, Taufkirchen, Germany) and b-FGF (basic fibroblast growth factor, PAN-Biotech, Aidenbach, Germany) at 5 µg/mL each. The cells were incubated at 37 °C and 5% CO_2_, and passaging took place twice a week or when the confluence reached about 80–90 percent.

#### 2.2.4. Direct Cytotoxicity Testing

For the direct cell culture tests, both the test specimens and reference materials (as described in Section 2.2.2.) were seeded with 240,000 cells/mL of cell culture medium in 12-well plates, placing the cells directly on the material surfaces. After a 24 h incubation under standard cell culture conditions, we conducted live/dead cell staining. This involved adding 60 μL of propidium iodide (PI) stock solution (concentration: 50 μg/mL PI in phosphate-buffered saline (PBS)) and 500 μL of freshly prepared fluorescein diacetate (FDA) working solution (concentration: 20 μg/mL in PBS, derived from a 5 mg/mL FDA in acetone stock solution) to each well. Following a short 3 min incubation at room temperature, the specimens were washed with pre-warmed PBS and immediately analyzed using the fluorescence microscope BZ-X800 (Keyence, Osaka, Japan) capable of simultaneous red and green fluorescence detection. The presence of green fluorescent cells in the microscopic field indicated living cells, while red fluorescent cells signified deceased cells.

#### 2.2.5. Indirect Cytotoxicity Testing

For indirect cell culture testing, we prepared extracts by incubating each specimen and toxic control samples in cell culture medium at a ratio of 3 cm^2^/mL for 72 h under standard cell culture conditions. As a negative control, cell culture medium alone was incubated similarly. Post-incubation, the specimens were removed, and the extracts were centrifuged at 14,000 RPM for 10 min. The resulting supernatants were then used for assay procedures.

In the assays, 96-well plates were seeded with 10,000 fibroblasts or keratinocytes per well in 100 μL of cell culture medium and incubated for 24 h. The medium was then replaced with 100 μL of the prepared extracts, and cells were incubated for an additional 24 h before proceeding to XTT and LDH assays.

For the XTT assay, we used the Cell Proliferation Kit II (XTT; Roche Diagnostics GmbH, Mannheim, Germany), following manufacturer instructions. It is based on the cleavage of the yellow tetrasodium salt (XTT) by viable cells to form a soluble orange formazan product that is directly proportional to the number of living cells. The assay involved mixing the electron-coupling reagent with XTT labeling reagent (1:50 dilution), adding 50 μL to the cells, incubating for 4 h, and then quantifying substrate conversion by measuring absorbance at 450 nm (650 nm reference) using an Enzyme Linked Immunosorbent Assay (ELISA) reader. Blank control absorbance values (medium without cells) were subtracted from the assay readings for accurate results.

For the LDH assay, the LDH-Cytotoxicity Assay Kit II (BioVision, Ilmenau, Germany) was used as according to the manufacturer’s guidelines. This process involved incubating 10 μL of cell supernatants with 100 μL LDH reaction mix for 30 min at room temperature, stopping the reaction, and measuring absorbance using an ELISA reader at 450 nm (650 nm reference). Blank control absorbance values (medium without cells) were subtracted from the assay readings for accurate results.

### 2.3. Wound Model

Human skin was obtained from patients undergoing plastic surgery at the Department of Plastic, Reconstructive and Aesthetic Surgery, University Medical Center Hamburg-Eppendorf. The patients gave their written consent to the anonymous use of their resected tissue. Surgical samples were immediately processed in the laboratory after being transported in holding media. The protocol used for sample preparation was similar to the approach of Wilkinson et al. [15] but with altered wound and explant diameter.

**Figure 1:**
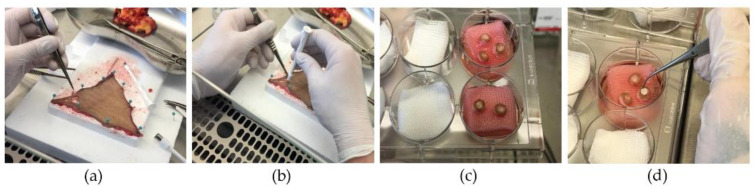
Fabrication of the wound model. (**a**) Subcutaneous fat is removed prior to fixation on a flat surface; (**b**) partial-thickness wounds created with a 3 mm punch followed by an 8 mm punch to harvest one sample; (**c**) introduction in cell culture medium while maintaining air–liquid interface (airlift); (**d**) implantation of silk matrices.

Figure 1 depicts the preparation of the wound model with the implantation of the silk matrices. 

#### 2.3.1. Skin Preparation and Wounding Procedure

The skin samples were disinfected using 70% ethanol (EtOH). After rinsing with PBS supplemented with 100 U/mL penicillin/streptomycin, fat tissue was carefully removed using surgical scissors. To prevent dehydration, additional rinsing with PBS was carried out.

For the wounding procedure, the skin was fixed onto a sterilized cutting board, ensuring the outer layer faced upwards. A 3 mm biopsy punch was employed to create a partial thickness wound, removing the middle piece with tweezers and a scalpel. This was followed by using an 8 mm biopsy punch around the initial wound.

#### 2.3.2. Introduction of Silk Wound Matrices

Before incubating the skin punches, two different silk wound matrices of 100 µm width and 3 mm diameter, either in the form of membranes or nonwovens, were press-fitted into the wound. Skin punches with no matrix applied served as control. For each application type (membrane, nonwoven, control) and day of analysis, 6 specimens were prepared.

#### 2.3.3. Incubation and Maintenance

A metal grid (airlift) covered with a nylon filter membrane was positioned in a 6-well plate. A serological pipette was used to introduce approximately 5.5 mL of medium (comprising 89% DMEM, 10% fetal calf serum (FCS), and 1% penicillin/streptomycin) per well. The punched-out skin samples were then accurately transferred onto the filter membrane. Partial-thickness, wounded skin specimens were cultured, maintaining their surface at the air–liquid interphase inside a cell incubator at 37° C and 5% CO_2_. The media were changed every three days.

### 2.4. Histological Analysis

This study focused on the utilization of hematoxylin and eosin (HE) staining to assess tissue architecture and monitor the healing process within the wound. Measurement of the wound closure was conducted using ImageJ software (ImageJ 1.54d, Java 1.8.0_345, Wayne Rasband and contributors, National Institutes of Health, Bethesda, MD, USA), while Ki67 staining was employed to evaluate cellular proliferation, and the TUNEL assay was utilized to visualize apoptotic activity.

#### 2.4.1. Hematoxylin and Eosin Staining

After incubation periods of 5, 10, 15, and 20 days, specimens were collected, fixed in 4% formalin, and embedded in paraffin for subsequent histological and immune histochemical evaluations. Sections (3.0 μm thick) from the wound center of paraffin-embedded samples were subjected to hematoxylin and eosin (HE) staining, following standardized protocols from the Institute for Osteology and Biomechanics (IOBM) at the University Medical Center Hamburg-Eppendorf, Germany. The protocol involved rehydration using graded ethanol and xylene solutions, and distilled water. This was followed by the application of hematoxylin for 10 min and 0.5% eosin for 6.5 min with intermediate rinsing in tap water. Finally, dehydration was performed using graded ethanol and xylene solutions. Images were acquired on the fluorescence microscope BZ-X800 (Keyence, Osaka, Japan) using its brightfield channel and further analyzed with ImageJ software.

#### 2.4.2. Immune Histochemical Staining

Analyses of both apoptosis and proliferation within the tissue sections were conducted. For apoptosis analysis, we used the TUNEL assay-kit (Abcam, Hamburg, Germany) to detect DNA fragmentation, a hallmark of apoptosis, in the wound tissues. This approach aimed to assess the level of programmed cell death in response to different treatments. To gain insights into the proliferative activity during re-epithelialization, tissue sections were immuno-stained for Ki67 (Abcam, Hamburg, Germany). Ki67 antibodies were used to assess keratinocyte proliferation and to evaluate the influence of the test materials on cell proliferation. This test particularly focused on examining cell proliferation close to the silk materials, offering a comprehensive view of the cellular dynamics in the wound healing process.

Preparation: Serial sections of paraffin-embedded samples, each of 3.0 μm thickness, were prepared and placed on microscope slides. The samples were deparaffinized using graded ethanol and xylene solutions before the histochemical staining process.

#### 2.4.3. Proliferation Analysis (Ki67)

Prior to antibody staining, sections were prepared following the manufacturer’s instructions from the IHC Detection Kit (Mouse and Rabbit Specific HRP/AEC Detection IHC Kit, ab93705; Abcam, Hamburg, Germany). After initial hydrogen peroxide blocking and washing in tris-buffered solution (TBS), the samples underwent antigen retrieval in citrate buffer (Citra Plus Solution; Biogenex Laboratories, Fremont, CA, USA), followed by incubation at 60 °C for 24 h. After rinsing, the application of protein-blocking reagent and subsequent washing in TBS/distilled water, the sections were stained with Ki67 antibody (LOT No. GR3351605-26, Abcam, Hamburg, Germany) at a dilution of 1:250. Sections without primary antibodies served as controls. Following the protocol of the detection kit, a biotinylated secondary antibody, streptavidin-peroxidase and AEC substrate (3-Amino-9-Ethylcarbazole) were applied after 24 h. Ki67 staining did not involve counterstaining. The sections were further subjected to statistical analysis.

#### 2.4.4. Apoptosis Analysis (TUNEL Assay-Kit)

Following deparaffinization, sections were processed with the TUNEL assay-kit using the In Situ Apoptosis Detection Kit ab206386 (Abcam, Hamburg, Germany) as per the manufacturer’s guidelines. In short: Terminal deoxynucleotidyl transferase (TdT) was used to add biotin-labeled deoxynucleotides to the 3′-OH ends of fragmented DNA. These biotinylated nucleotides were then bound with a streptavidin-horseradish peroxidase (HRP) conjugate. Diaminobenzidine (DAB) reacted with the HRP to produce an insoluble brown substrate at the sites of DNA fragmentation. Methyl green served as the counterstain. This analysis method was used primarily for visualization purposes, aiming to qualitatively assess the presence or absence of apoptosis-inducing effects associated with the silk biomaterial.

### 2.5. Statistical Analysis

First, a descriptive presentation of all relevant parameters was performed as part of the statistical analysis. The count and frequency were specified for categorical variables, while the arithmetic mean and standard deviation were calculated for metric variables. Further analysis using statistical models was carried out using mixed linear regression.

Wound regeneration (epithelial coverage in percent) and proliferation (as absolute Ki67 counting) were used as outcome parameters. The surface was set as a fixed factor—modeled as type 3 effects—in the statistical model. The time course was modeled using a mixed effect with a 1st order autoregressive covariance structure. All statistical tests were performed at a type I error of alpha = 0.05—consequently, all results with a *p*-value of *p* < 0.05 were considered to be statistically significant. Due to the exploratory nature of the study, no adjustment was made for multiple testing between the two outcome parameters. 

The statistical software “IBM^®^ SPSS^®^ Statistics” version 25 was used for data collection, data processing and data analysis.

In HE-stained sections, the wound length and the length of the regenerating epithelial layer were quantified using ImageJ software. The percentage of wound recovery was determined by the following equation:

$$Wound\;Recovery\;\left(\%\right)=\left(\frac{Regenerating\;Epithelial\;Length}{Wound\;Length}\right)\times 100$$

In Ki67 immune histochemistry analysis, a region of interest (ROI) was defined corresponding to the wound area. Given the variability in wound sizes across samples, the ROI dimensions were not consistent but tailored to each sample’s wound region. Cells immune-positive for Ki67 within the ROI were manually counted.

## 3. Results

### 3.1. Biological Evaluation

**Figure 2:**
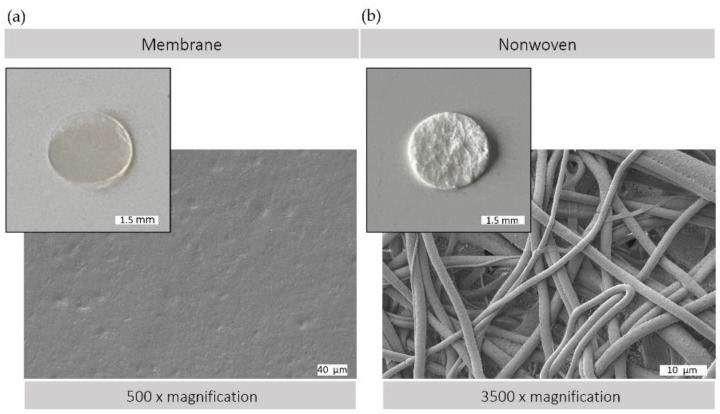
Silk matrices. Macroscopic and scanning electron (SEM) microscopic view of (**a**) silk fibroin membrane surface (500× magnification) and (**b**) silk fibroin nonwoven surface (3500× magnification); SEM image: Crossbeam 340, Zeiss, Oberkochen, Germany; prior to observation, samples were coated with a thin layer of gold via sputter deposition.

The morphological versatility of silk fibroin matrices allows them to be used in a variety of forms for wound regeneration. Figure 2 illustrates the structural characteristics of the silk fibroin matrices used, both membranes and nonwovens, as observed by macroscopic and electron microscopic analysis. Membranes produced by the casting process have a comparatively smooth and flat surface with minimal roughness, providing a predominantly two-dimensional surface that is conducive to cell adhesion and proliferation. In contrast, electrospun nonwovens have a three-dimensional architecture with varying degrees of porosity. This three-dimensional scaffold consists of interconnected fibers, providing a large surface area with spaces between, which can facilitate enhanced cell infiltration and colonization. In addition, the nonwoven structure provides a greater surface area to volume ratio, which could promote improved diffusion and cell exchange within the cellular microenvironment.

The first step of the biological evaluation was to conduct a confirmatory assessment of the biocompatibility of silk fibroin membranes and nonwovens with L929 mouse fibroblasts and HaCaT immortalized human keratinocytes, following the German version of the EN ISO 10993-5 standards [31]. This step served as a proof of concept to reaffirm known biocompatibility profiles, ensuring a solid foundation for the project’s further investigations.

A combination of direct and indirect contact assays with fibroblasts and keratinocytes was used, to assess biocompatibility. As per the guideline, a reduction in cell viability exceeding 30% of the negative control was interpreted as a cytotoxic effect. Furthermore, the assessment of interaction between cells and test materials was performed after 24 h of direct contact cultivation.

#### 3.1.1. Indirect Testing

**Figure 3:**
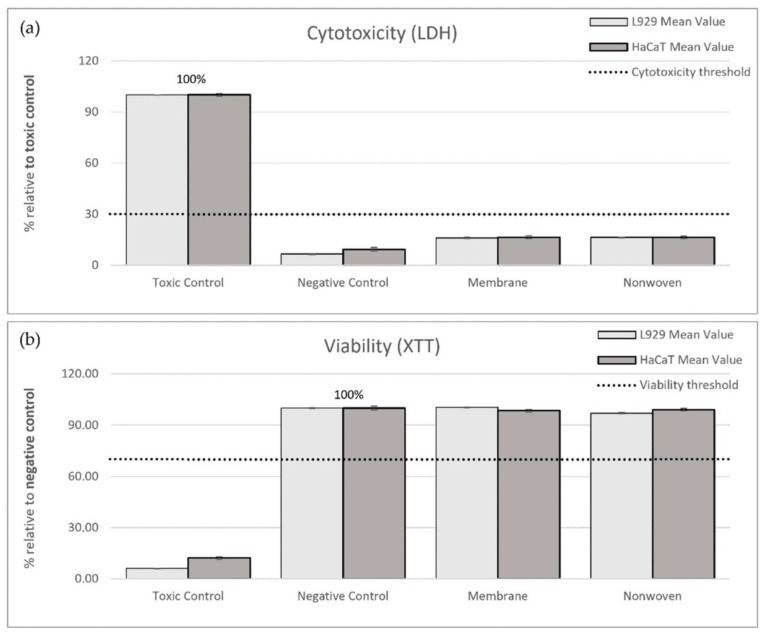
Results of indirect cytotoxicity testing of silk fibroin membranes and nonwoven materials. Cell lines used: L929 fibroblasts and HaCaTs. (**a**) Cytotoxicity was assessed by measuring lactate dehydrogenase (LDH) release after 24 h exposure to extracts from different materials. ELISA readings from the toxic control (RM-A) were used as the benchmark, representing 100% cytotoxicity. (**b**) Cell viability was measured using the XTT assay, which detects the metabolism of XTT to water-soluble, orange-colored formazan salt by viable cells. ELISA readings from the negative control (culture medium) were set as the benchmark, representing 100% viability. The measured values are expressed as percentages relative to the toxic control (LDH) or negative control (XTT). The shown percentages are mean values ± SEM.

In the LDH cytotoxicity assay, the ELISA readings from the toxic control (RM-A) served as a benchmark representing 100% cytotoxicity. Relative to this control, the membrane samples exhibited 16.13% cytotoxicity for fibroblasts and 16.54% for keratinocytes, while the nonwoven samples showed 16.45% and 16.49% cytotoxicity, respectively, all significantly below the 30% threshold indicating non-cytotoxic behavior (Figure 3a).

For viability, the negative control, representing normal cell growth, was set as the 100% viability benchmark. Post-exposure to the membrane, viability readings for fibroblasts and keratinocytes were 100.31% and 98.43%, respectively, and for the nonwoven, they were 96.95% and 99.08%. These values not only exceeded the 70% viability threshold but also approached or slightly surpassed the benchmark set by the negative control, affirming the non-toxic nature of the materials (Figure 3b).

In summary, the cytocompatibility of silk fibroin membranes and nonwovens was substantiated by both low cytotoxicity and high viability indices in comparison to the respective toxic and negative controls.

#### 3.1.2. Direct Testing

**Figure 4:**
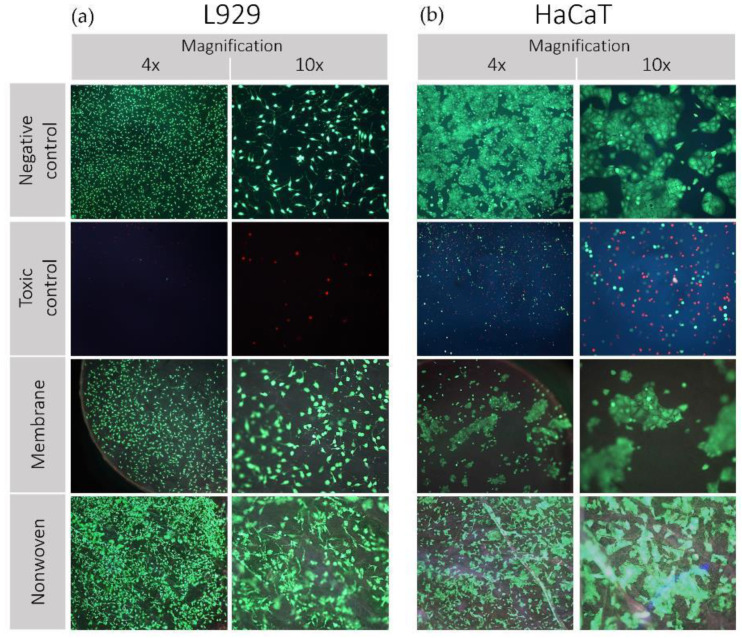
Results of direct cytotoxicity testing (live/dead staining) of silk fibroin membranes and nonwoven. Cell lines used: (**a**) L929 mouse fibroblasts and (**b**) HaCaT (human immortalized keratinocytes). Propidium iodide (PI) was employed to intercalate with the nucleic acids of dead cells, eliciting red fluorescence as an indicator of cell death. Meanwhile, fluorescein diacetate (FDA) was utilized to be hydrolyzed by esterases in living cells with intact membranes, resulting in green fluorescence, indicative of cell viability. These dyes were selected to differentiate between live (green) and dead (red) cells. Images were captured using a fluorescence microscope at various magnifications.

The vitality and adhesion behavior of both cell lines on silk fibroin membranes and nonwovens is shown in Figure 4.

L929 Fibroblasts: Analysis by fluorescent light revealed a predominantly viable population of fibroblasts on the fibroin membranes, nonwovens, and the negative control, characterized by a preponderance of green staining (Figure 4a). The spindle-shaped morphology of the fibroblasts remained intact, and the little to no occurrence of red-stained cells across the samples reinforced the non-toxic nature of the silk fibroin materials. In contrast, the positive toxic control displayed minimal cell attachment, with the few cells observed exhibiting a rounded morphology indicative of compromised viability.

HaCaT Keratinocytes: Similarly, keratinocytes on the negative control maintained their distinct clustering behavior and morphology, with no evidence of red staining, indicating high viability (Figure 4b). Observations of the membrane and nonwoven samples showed a slightly reduced density but still substantial cell clustering, upholding typical cellular morphology indicative of robust adhesion and viability. The toxic control samples were characterized by a scarce number of rounded cells, confirming the adverse conditions, and aligning with expectations of a cytotoxic environment. The preservation of cell morphology and the absence of significant cell death indicate the suitability of the material for biomedical applications involving direct cell–material contact.

### 3.2. Evaluation of the Ex Vivo Wound Healing

**Figure 5:**
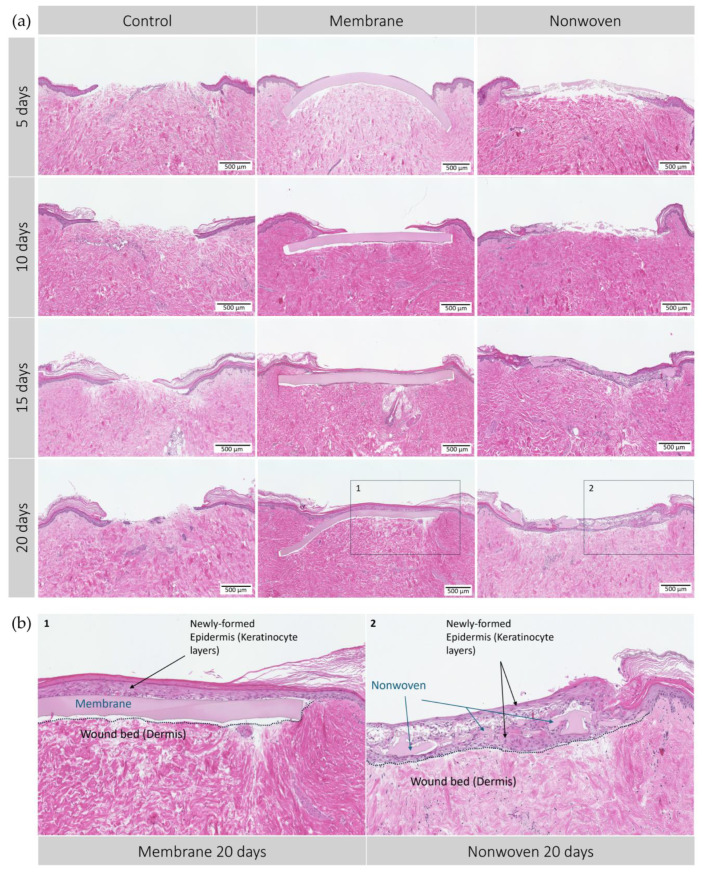
Hematoxylin–eosin (HE) staining of the human ex vivo wound model. Slices 3 µm thick from the center of the wound at different time points. After incubation period from 5 to 20 days with silk matrices, the samples were fixed in formalin and subjected to histological staining. (**a**) Display of each group and day; (**b**) detailed enlargement of the areas indicated in a: 1—membrane, 2—nonwoven. Dotted lines represent the extent of the wound bed. Scale bar indicates 500 µm. Untreated wounds served as controls.

In the ex vivo part of the analysis, the capacity for wound closure of epithelial tissue driven by the silk fibroin matrices was evaluated compared to untreated controls over 20 days. The membranes demonstrated an accelerated closure process, evidenced by early and progressive growth of epidermal cells on the matrix material from day 5 through day 20 (Figure 5).

The control group, lacking a wound matrix, presented a distinct healing dynamic. By day 20, these wounds were still partially open, likely due to the absence of a protective barrier similar to a blood clot-derived scab, which in vivo, would safeguard the wound and support the healing underneath.

Development of the epidermis in wounds treated with silk fibroin-based matrices showed notable differences. By day 5, membrane-treated wounds displayed varying degrees of epidermal re-epithelialization, with most showing the initial formation of epithelial monolayers, indicating a regenerative process. This regenerative trend continued, with the membrane-treated wounds showing remarkable integration of the matrices within the skin tissue and progressing cellular regeneration on its surface, most likely due to the membranes’ homogeneous microstructure predominantly supporting surface cell growth and early onset of wound closure. By days 10 and 15, complete wound closure was observed in most samples, with the epidermal layer visibly thickening over time to display a healthy, uniform skin structure without signs of excessive apoptosis or necrosis. By day 20, a well-formed keratinized layer indicated mature epithelial healing (Figure 5b).

In contrast, wounds treated with nonwoven matrices presented a different healing trajectory from the outset, showing less growth on the surface and more cellular infiltration, leading to patchy monolayers of epidermal cells across the nonwovens. The fibrous and porous nature of the nonwoven matrix facilitated this cellular infiltration, observed as early as day 5. Over time, cells not only permeated the matrix but also proliferated beneath it, fostering a dense and integrative healing environment. By day 20, regeneration led to complete wound closure, characterized by full integration of the cells within the material and the formation of a multi-layered epidermal structure, although less uniform than that observed in the membrane-treated wounds (Figure 5b).

**Figure 6:**
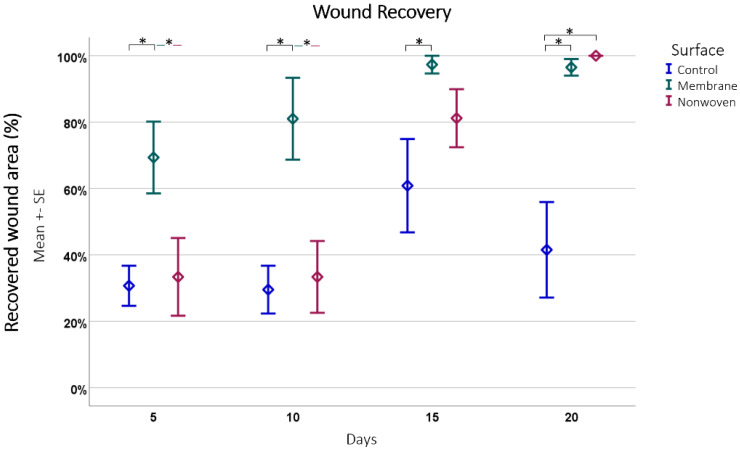
Wound recovery by epithelial coverage. This figure depicts the progression of epithelial coverage over time (20 days), expressed as a percentage of the original wound length. The data were obtained from hematoxylin and eosin (HE)-stained samples subsequently analyzed using ImageJ software. Results are presented as means ± SEM, based on six observations (*n* = 6). Square brackets indicate significance compared to control. Colored horizontal lines represent significant differences between the samples. Asterisks (*) denote statistical significance at *p* < 0.05.

For quantitative evaluation, the total wound size was measured, and the extent of wound coverage by re-epithelialization was quantified, with the results expressed as a percentage of the original wound length (Figure 6).

The analysis showed that fibroin membranes were the most effective in promoting regeneration, with an average of 86.04% regenerated epithelium, significantly outperforming the nonwoven group at 61.96% (*p* = 0.0008) and the control group at 40.63% (*p* < 0.0001) at all time points and the difference between the nonwoven was also significant to the control (*p* = 0.0026).

A closer look at each material at different time points revealed distinct recovery patterns (Figure 6). The membrane-treated wounds consistently showed an increase in epithelial coverage, starting from 69.31% on day 5 to nearly full coverage by day 15, and maintaining a high level of coverage by day 20. The nonwoven group, while starting at a lower coverage of 33.42% on day 5, showed significant improvement by day 15 and achieved full coverage by day 20. Simultaneously, cellular infiltration of the matrix occurs, which should have an influence on its integration and later degeneration. In contrast, the control group wounds had the least effective recovery, starting at 30.54% coverage on day 5 and showing fluctuations in coverage over time, ultimately reducing by day 20.

Comparing the materials at each time point further highlighted the consistent growth stimulation of the fibroin membrane. Significant improvements in regeneration were noted for it compared to the control, with *p*-values ranging from 0.0060 to 0.0001. In the later stages, the nonwoven also demonstrated significant improvements over the control, especially noticeable on days 15 and 20. In both cases the treated wounds displayed nearly complete or complete closure, in contrast to the control wounds, which often showed less than 50% closure.

**Figure 7:**
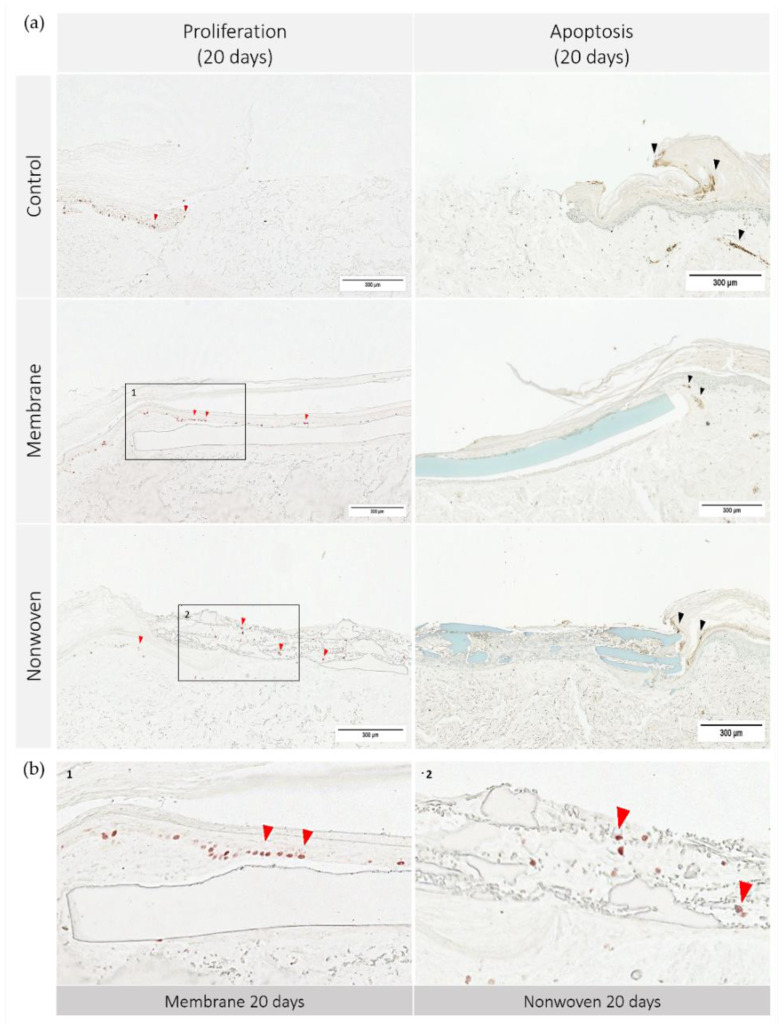
Cell apoptosis and proliferation in human ex vivo wound model. Slices 3 µm thick from the center of the wound at day 20. Immunohistochemical staining with Ki67 antibodies for proliferation (no counterstain) and TUNEL assay-kit for apoptosis (methyl green counterstain). Ki67 staining highlights actively dividing cells, resulting in red stained cell nuclei, while TUNEL assay detects DNA fragmentation characteristic of apoptotic cells, seen as dark brown areas. (**a**) Display of each group on day 20; (**b**) detailed enlargement of the areas indicated in a: 1—membrane, 2—nonwoven; red arrowhead: example of Ki67 positive cells, black arrowhead: example of apoptotic cells; scale bar indicates 300 µm.

To further elucidate the mechanisms underlying skin regeneration in our model, we conducted analyses of both apoptosis and proliferation within the tissue sections. Figure 7 illustrates the apoptosis and proliferation activity on day 20.

**Figure 8:**
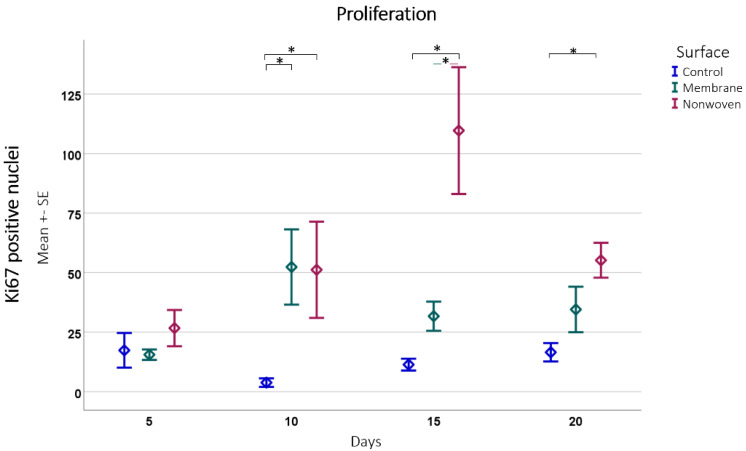
Proliferation detected by Ki67 positive cell counting subsequently analyzed using ImageJ software. Results are presented as means ± SEM, based on six observations (*n* = 6). Square brackets indicate significance compared to control. Colored horizontal lines represent significant differences between the samples. Asterisks (*) denote statistical significance at *p* < 0.05.

The immune histochemical assessment of the wound model revealed distinct interactions between keratinocytes and the two types of fibroin-based matrices although in both cases the keratinocytes had spread over the entire skin defect. In the membrane-treated samples, proliferating keratinocytes were frequently observed in proximity to and on the surface of the membrane, suggesting that it provided a favorable scaffold for cellular expansion (Figure 7b). This was not merely an isolated observation but was substantiated by statistical findings showing a significant increase in Ki67 immune-positive cells compared to the control, particularly notable on day 10 (*p* = 0.0054 for membrane vs. control; Figure 8).

In the nonwoven-treated wounds, a high concentration of proliferating keratinocytes was found within and beneath the silk fibroin matrices, which could be attributed to the high surface-to-volume ratio enhancing cell adhesion and proliferation. The nonwoven group displayed a substantial increase in proliferative cells especially on day 15, which was significantly higher than both the control (*p* < 0.0001) and the membrane group (*p* < 0.0001). By day 20, although the number of proliferating cells in the nonwoven group slightly decreased, it remained significantly higher than the control group, maintaining a robust proliferative response (Figure 8).

The observation of apoptosis unveiled a trend that paralleled the proliferation data: wounds with a higher number of proliferating cells, such as the nonwoven-covered ones also tended to exhibit slightly increased apoptosis. However, this phenomenon was uniform in all groups, including untreated controls, indicating a balanced and consistent pattern of cell turnover. Notably, there were no regions with exceptionally high apoptosis in any of the samples.

## 4. Discussion

Silk fibroin as a biomaterial for wound healing was tested here as a potential alternative to conventional approaches, aiming to address the limitations and drawbacks associated with current materials. In this study, we confirmed the excellent biocompatibility of silk fibroin matrices and evaluated their regenerative potential using a human 3D ex vivo wound model. We quantitatively analyzed their beneficial impact on keratinocyte activity and the overall dynamics of wound healing. Our previous studies have already revealed that additive-free electrospun nonwovens [28] and formic acid/CaCl_2_-derived silk fibroin membranes [29] exhibit high biocompatibility and regenerative properties supporting fibroblast adhesion and proliferation. In here, biocompatibility was affirmed through both direct and indirect assays, indicating viability levels that closely mirrored or surpassed control conditions, with the membranes and nonwovens showing low cytotoxic levels well below the 30% threshold. These findings align with a study by Luangbudnark W. et al. 2012 where biocompatibility of chitosan and silk fibroin blended films were tested through a period of 14 days, showing no differences to the control group regarding XTT assay [32]. The group of Zhang et al. 2017 had similar findings subjecting silk fibroin films to a cytotoxicity test with mouse fibroblasts L-929 cells [11]. In addition, normal human dermal fibroblast (NHDF) cells remained viable in a live/dead assay, when seeded on electrospun silk fibroin membranes, with their number increasing along time [33].

The data from histological evaluation highlight the significant impact of silk fibroin matrices on skin regeneration and wound healing. Throughout the observation, membranes consistently demonstrated effective tissue stimulation leading to complete wound regeneration within 20 days with a cumulative coverage of 86.04%. Similarly, nonwovens showed beneficial effects, especially visible in the later stages of the healing process with complete wound closure. However, a more homogenous epithelial layer was formed on top of fibroin membranes, while nonwovens exhibited pronounced cell infiltration with less uniform layers. These findings reveal that wounds treated with fibroin-based matrices, like membranes or nonwovens, exhibited markedly improved outcomes in terms of re-epithelialization and wound closure compared to untreated tissue. This supports the effectiveness of silk-based wound matrices in this model, indicating their significant role in enhancing the process of skin regeneration. The effects on proliferation and support of wound closure in ex vivo and in vitro experiments are well documented [10,11,18,25,34,35,36]. For instance, acceleration in wound healing due to fibroin films was observed in an in vivo study comparing them to commercially available wound dressings in full-thickness skin defects of rabbits [11]. 

Considering the microstructural influence of silk fibroin matrices on wound healing, our study shows distinct surface interactions with cells. Silk fibroin membranes predominantly offer a two-dimensional surface facilitating cellular attachment, whereas nonwovens provide a three-dimensional nanofibrous matrix, allowing cell infiltration. The membrane’s smoother surface promoted rapid cellular coverage (69.31% by day 5), while the nonwoven’s nanofibrous structure supported not only cell adhesion but also an increase in cell proliferation: Proliferation peaked at 109.67 cells on day 15. This suggests that the nonwoven’s architecture is instrumental in promoting an optimal regenerative environment. Our observations are in line with the findings of Li et al. 2016, where structural variations (with and without a fibrous network) in silk fibroin scaffolds significantly influenced dermal wound healing in rats [37]. In addition, scaffolds that mimic the extracellular matrix, which is primarily composed of fibrous proteins and glycosaminoglycans, have been shown to have good regenerative properties [38]. The apoptosis pattern remained consistent across all analyses. This consistency in apoptotic activity suggests that while the fibroin matrices effectively stimulated cell proliferation, they did not negatively influence the rate of programmed cell death in the tissues. It supports the assumption that fibroin matrices aid in wound regeneration without disturbing the skin’s natural balance of cellular apoptosis and proliferation.

Given the limitations of this ex vivo setting, no degradation of the silk material was observed. Apparent structural losses, particularly in the nonwoven samples, were artifacts resulting from the sample preparation process. Actual degradation was unlikely, as the biodegradation of fibroin materials in vivo typically requires an immune response involving macrophages or other phagocytic cells [39]. The in vivo degradation timelines for silk fibroin materials vary from 5 weeks [40] to 12 months [22], influenced by the material’s processing, structure, and implantation site, as well as the accessibility to immune cells [39,41]. The absence of a fully active immune system in our ex vivo model likely explains the lack of observed degradation and absence of inflammatory signs. However, ex vivo human skin contains resident immune cells as shown in previous studies [42,43,44]; nonetheless, this should only affect the first few days of the analyses, as the life span of these cells is a maximum of four days.

Full- or partial-thickness wounds created in ex vivo human skin models, whether by scalpel or punch biopsy, typically re-epithelialize completely, as seen in previous studies [15,16,45]. However, in our study, the control groups exhibited an average recovery less than 50% summed over all time points, with only a minority of samples showing full recovery at later stages. This variance could be attributed to differences in wound size and depth or variations in culture conditions. Less regeneration has also been observed in untreated controls versus electrospun silk fibroin scaffolds [18]. Additionally, the absence of a wound scab might have impeded healing, since wound scabs offer protection against dehydration and provide a platform for cellular proliferation [46]. A further limitation of this study refers to the used abdominoplasties. The donor skin is individual; the tissue quality and therefore also the healing potential varies. As the donors are anonymous, it is not possible to rule out the presence of diet-induced diabetes, which patients who undergo abdominoplasty often have. This certainly also influences the proliferation and migration of skin cells.

## 5. Conclusions

In this study the effectiveness of silk fibroin membranes and nonwovens regarding skin wound healing was evaluated. The morphological versatility of the silk fibroin materials allows them to be used in various forms for wound regeneration. Data presented suggest that fibroin matrices, particularly the nonwovens, can significantly enhance keratinocyte proliferation and migration. An ingrowth of the material could be shown, which should be advantageous for deeper wounds (e.g., pressure ulcers including the subcutis) in particular when biodegradation has already been proven to occur after 3–12 weeks. When using the fibroin membrane in human ex vivo wounds, it was shown that the microstructure and the material act as a perfect “splint” for the keratinocytes. Tongue-like, they expand rapidly on its surface and ensure wound closure. These translational results with human skin now only require in vivo confirmation for these partial thickness wounds.

Additionally, the proven biodegradation is a superior feature compared to matrices already on the market, as a stable wound closure remains after dissolution. These outcomes support the efficacy of fibroin materials in wound healing making it a preferable option in clinical settings. The effectiveness of using the human ex vivo wound model for evaluating wound dressing materials was demonstrated. The human ex vivo 3D wound model is a valuable tool for analyzing wound care materials, techniques, and products. Its vitality over 3 weeks makes it possible to draw translational conclusions about human in vivo reactions, as human skin differs significantly from animal skin. It is an important intermediate step towards in vivo analysis and can reduce animal testing.

Future research should focus on conducting in vivo studies to confirm the translational potential of silk fibroin matrices observed in the ex vivo model. Exploring modifications to the silk fibroin matrices, such as incorporating growth factors or other bioactive agents, could further enhance their regenerative properties. Additionally, developing protocols for the clinical application of biodegradable silk fibroin matrices in various types of wounds and assessing the cost-effectiveness and practicality of using silk fibroin-based dressings in clinical settings compared to existing wound care products are important next steps.

## Data Availability

The data presented in this study are available upon request from the corresponding author. The data are not publicly available due to ownership rights held by the University Medical Center Hamburg-Eppendorf, 20246 Hamburg, Germany.

## References

[1] Patil P.P., Reagan M.R., Bohara R.A. (2020). Silk fibroin and silk-based biomaterial derivatives for ideal wound dressings. Int. J. Biol. Macromol..

[2] Broussard K.C., Powers J.G. (2013). Wound dressings: Selecting the most appropriate type. Am. J. Clin. Dermatol..

[3] Farokhi M., Mottaghitalab F., Fatahi Y., Khademhosseini A., Kaplan D.L. (2018). Overview of Silk Fibroin Use in Wound Dressings. Trends Biotechnol..

[4] Lehmann T., Vaughn A.E., Seal S., Liechty K.W., Zgheib C. (2022). Silk Fibroin-Based Therapeutics for Impaired Wound Healing. Pharmaceutics.

[5] Winkler S., Kaplan D.L. (2000). Molecular biology of spider silk. Rev. Mol. Biotechnol..

[6] Rockwood D.N., Preda R.C., Yücel T., Wang X., Lovett M.L., Kaplan D.L. (2011). Materials fabrication from *Bombyx mori* silk fibroin. Nat. Protoc..

[7] Mottaghitalab F., Farokhi M., Shokrgozar M.A., Atyabi F., Hosseinkhani H. (2015). Silk fibroin nanoparticle as a novel drug delivery system. J. Control. Release.

[8] Kamalathevan P., Ooi P.S., Loo Y.L. (2018). Silk-Based Biomaterials in Cutaneous Wound Healing: A Systematic Review. Adv. Ski. Wound Care.

[9] Gholipourmalekabadi M., Sapru S., Samadikuchaksaraei A., Reis R.L., Kaplan D.L., Kundu S.C. (2020). Silk fibroin for skin injury repair: Where do things stand?. Adv. Drug Deliv. Rev..

[10] Mazurek Ł., Szudzik M., Rybka M., Konop M. (2022). Silk Fibroin Biomaterials and Their Beneficial Role in Skin Wound Healing. Biomolecules.

[11] Zhang W., Chen L., Chen J., Wang L., Gui X., Ran J., Xu G., Zhao H., Zeng M., Ji J. (2017). Silk Fibroin Biomaterial Shows Safe and Effective Wound Healing in Animal Models and a Randomized Controlled Clinical Trial. Adv. Health Mater..

[12] Hasatsri S., Angspatt A., Aramwit P. (2015). Randomized Clinical Trial of the Innovative Bilayered Wound Dressing Made of Silk and Gelatin: Safety and Efficacy Tests Using a Split-Thickness Skin Graft Model. Evid. Based Complement. Altern. Med..

[13] Stone R., Wall J.T., Natesan S., Christy R.J. (2018). PEG-Plasma Hydrogels Increase Epithelialization Using a Human Ex Vivo Skin Model. Int. J. Mol. Sci..

[14] De Wever B., Kurdykowski S., Descargues P. (2015). Human Skin Models for Research Applications in Pharmacology and Toxicology: Introducing NativeSkin^®^, the “Missing Link” Bridging Cell Culture and/or Reconstructed Skin Models and Human Clinical Testing. Appl. Vitr. Toxicol..

[15] Wilkinson H.N., Kidd A.S., Roberts E.R., Hardman M.J. (2021). Human Ex vivo Wound Model and Whole-Mount Staining Approach to Accurately Evaluate Skin Repair. J. Vis. Exp..

[16] Xu W., Jong Hong S., Jia S., Zhao Y., Galiano R.D., Mustoe T.A. (2012). Application of a partial-thickness human ex vivo skin culture model in cutaneous wound healing study. Lab. Investig..

[17] Balaji S., Moles C.M., Bhattacharya S.S., LeSaint M., Dhamija Y., Le L.D., King A., Kidd M., Bouso M.F., Shaaban A. (2014). Comparison of interleukin 10 homologs on dermal wound healing using a novel human skin ex vivo organ culture model. J. Surg. Res..

[18] Hodgkinson T., Yuan X.-F., Bayat A. (2014). Electrospun silk fibroin fiber diameter influences in vitro dermal fibroblast behavior and promotes healing of ex vivo wound models. J. Tissue Eng..

[19] Bai S., Han H., Huang X., Xu W., Kaplan D.L., Zhu H., Lu Q. (2015). Silk scaffolds with tunable mechanical capability for cell differentiation. Acta Biomater..

[20] She Z.-d., Liu W.-q., Feng Q.-l. (2009). Preparation and cytocompatibility of silk fibroin/chitosan scaffolds. Front. Mater. Sci. China.

[21] Guang S., An Y., Ke F., Zhao D., Shen Y., Xu H. (2015). Chitosan/silk fibroin composite scaffolds for wound dressing. J. Appl. Polym. Sci..

[22] Wang Y., Rudym D.D., Walsh A., Abrahamsen L., Kim H.J., Kim H.S., Kirker-Head C., Kaplan D.L. (2008). In Vivo degradation of three-dimensional silk fibroin scaffolds. Biomaterials.

[23] Horan R.L., Antle K., Collette A.L., Wang Y., Huang J., Moreau J.E., Volloch V., Kaplan D.L., Altman G.H. (2005). In vitro degradation of silk fibroin. Biomaterials.

[24] Kopp A., Smeets R., Gosau M., Friedrich R.E., Fuest S., Behbahani M., Barbeck M., Rutkowski R., Burg S., Kluwe L. (2019). Production and Characterization of Porous Fibroin Scaffolds for Regenerative Medical Application. In Vivo.

[25] Chouhan D., Mandal B.B. (2020). Silk biomaterials in wound healing and skin regeneration therapeutics: From bench to bedside. Acta Biomater..

[26] Wani S.U.D., Zargar M.I., Masoodi M.H., Alshehri S., Alam P., Ghoneim M.M., Alshlowi A., Shivakumar H.G., Ali M., Shakeel F. (2022). Silk Fibroin as an Efficient Biomaterial for Drug Delivery, Gene Therapy, and Wound Healing. Int. J. Mol. Sci..

[27] Pastar I., Liang L., Sawaya A.P., Wikramanayake T.C., Glinos G.D., Drakulich S., Chen V., Stojadinovic O., Davis S.C., Tomic-Canic M., Marques A.P., Pirraco R.P., Cerqueira M.T., Reis R.L. (2018). 10—Preclinical models for wound-healing studies. Skin Tissue Models.

[28] Kopp A., Smeets R., Gosau M., Kröger N., Fuest S., Köpf M., Kruse M., Krieger J., Rutkowski R., Henningsen A. (2020). Effect of process parameters on additive-free electrospinning of regenerated silk fibroin nonwovens. Bioact. Mater..

[29] Kopp A., Schunck L., Gosau M., Smeets R., Burg S., Fuest S., Kröger N., Zinser M., Krohn S., Behbahani M. (2020). Influence of the Casting Concentration on the Mechanical and Optical Properties of FA/CaCl_2_-Derived Silk Fibroin Membranes. Int. J. Mol. Sci..

[30] Fuest S., Salviano-Silva A., Maire C.L., Xu Y., Apel C., Grust A.L.C., Delle Coste A., Gosau M., Ricklefs F.L., Smeets R. (2024). Doping of casted silk fibroin membranes with extracellular vesicles for regenerative therapy: A proof of concept. Sci. Rep..

[31] (2012). Biological Evaluation of Medical Devices—Part 5: Tests for In Vitro Cytotoxicity.

[32] Luangbudnark W., Viyoch J., Laupattarakasem W., Surakunprapha P., Laupattarakasem P. (2012). Properties and biocompatibility of chitosan and silk fibroin blend films for application in skin tissue engineering. Sci. World J..

[33] Miguel S.P., Simões D., Moreira A.F., Sequeira R.S., Correia I.J. (2019). Production and characterization of electrospun silk fibroin based asymmetric membranes for wound dressing applications. Int. J. Biol. Macromol..

[34] Zhang Y., Lu L., Chen Y., Wang J., Chen Y., Mao C., Yang M. (2019). Polydopamine modification of silk fibroin membranes significantly promotes their wound healing effect. Biomater. Sci..

[35] Guo P., Du P., Zhao P., Chen X., Liu C., Du Y., Li J., Tang X., Yang F., Lv G. (2021). Regulating the mechanics of silk fibroin scaffolds promotes wound vascularization. Biochem. Biophys. Res. Commun..

[36] Schneider A., Wang X.Y., Kaplan D.L., Garlick J.A., Egles C. (2009). Biofunctionalized electrospun silk mats as a topical bioactive dressing for accelerated wound healing. Acta Biomater..

[37] Li X., You R., Luo Z., Chen G., Li M. (2016). Silk fibroin scaffolds with a micro-/nano-fibrous architecture for dermal regeneration. J. Mater. Chem. B.

[38] Kim T.G., Shin H., Lim D.W. (2012). Biomimetic Scaffolds for Tissue Engineering. Adv. Funct. Mater..

[39] Thurber A.E., Omenetto F.G., Kaplan D.L. (2015). In vivo bioresponses to silk proteins. Biomaterials.

[40] Zhou J., Cao C., Ma X., Hu L., Chen L., Wang C. (2010). In vitro and in vivo degradation behavior of aqueous-derived electrospun silk fibroin scaffolds. Polym. Degrad. Stab..

[41] Sengupta S., Park S.-H., Seok G.E., Patel A., Numata K., Lu C.-L., Kaplan D.L. (2010). Quantifying Osteogenic Cell Degradation of Silk Biomaterials. Biomacromolecules.

[42] Rakita A., Nikolić N., Mildner M., Matiasek J., Elbe-Bürger A. (2020). Re-epithelialization and immune cell behaviour in an ex vivo human skin model. Sci. Rep..

[43] He X., de Oliveira V.L., Keijsers R., Joosten I., Koenen H.J. (2016). Lymphocyte Isolation from Human Skin for Phenotypic Analysis and Ex Vivo Cell Culture. J. Vis. Exp..

[44] Dijkgraaf F.E., Matos T.R., Hoogenboezem M., Toebes M., Vredevoogd D.W., Mertz M., van den Broek B., Song J.Y., Teunissen M.B.M., Luiten R.M. (2019). Tissue patrol by resident memory CD8^+^ T cells in human skin. Nat. Immunol..

[45] Kratz G. (1998). Modeling of wound healing processes in human skin using tissue culture. Microsc. Res. Technol..

[46] Phillips S.J. (2000). Physiology of wound healing and surgical wound care. Asaio J..

